# Bioinspired Computation and Its Applications in Operation Management

**DOI:** 10.1155/2014/356571

**Published:** 2014-06-12

**Authors:** Tinggui Chen, Jianjun Yang, Kai Huang, Qiang Cheng

**Affiliations:** ^1^School of Computer and Information Engineering, Zhejiang Gongshang University, Hangzhou 310018, China; ^2^Department of Computer Science, University of North Georgia, Oakwood, GA 30566, USA; ^3^DeGroote School of Business, McMaster University, DSB 404, Hamilton, ON, Canada L8S 4M4; ^4^College of Mechanical Engineering and Applied Electronics, Beijing University of Technology, Beijing 100124, China

Bioinspired computation is an umbrella term for different computational technologies that are based on principles or models of biological systems. This class of approaches, including evolutionary algorithm, swarm intelligence, and artificial immune system, complements traditional ones in the sense that the former can be applied to large and complex combination optimization problems, but the latter encounters difficulties. Therefore, bioinspired technologies are becoming important in the face of solving discrete and dynamic problems.

Recently, the bioinspired computation has attracted much attention of researchers and has also been widely applied to operation management fields ranging between production assembling, inventory control, project scheduling, human resource management, and revenue management. However, due to complexity and uncertainty in operation management problems, it is very difficult to find out the optimum solution under the limited resources, time, and money in real-world applications using the bioinspired technologies. Therefore, it is necessary to develop efficient or improved algorithms to solve operation management problems.

The main objective of this special issue is to present the original research and review articles on the latest theoretical and practical achievements that will contribute to the field of bioinspired computation and its applications in operation management, in all branches of management science and computer science.

The special issue received 66 high-quality submissions from different countries all over the world. All submitted manuscripts have followed the same standard (peer-reviewed by at least three independent reviewers) as applied to regular ones to “this journal.” Due to space limit, only 24 papers could be published (acceptance ratio of 1 : 3). Inevitably, difficult decisions had to be made, and some high-quality submissions could not be included. The primary guideline was to demonstrate the wide scope of bioinspired computation and applications in operation management. Besides, some novel research questions from different applications that are worth further investigation in the future are also included.

In the paper, “*A hybrid genetic-simulated annealing algorithm for the location-inventory-routing problem considering returns under e-supply chain environment*,” Y. Li et al. formulate a location-inventory-routing problem model with no quality defects' returns. In addition, to solve this NP-hard problem, an effective hybrid genetic-simulated annealing algorithm (HGSAA) is proposed. Results of numerical examples show that HGSAA outperforms GA in computing time, optimal solution, and computing stability. Moreover, the proposed model is also very useful in helping managers make the right decisions under e-supply chain environment.

In the paper “*Optimization and planning of emergency evacuation routes considering traffic control,*” G. Li et al. establish two different emergency evaluation models on the basis of the maximum-flow model (MFM) and minimum-cost maximum-flow model (MC-MFM) and provide the corresponding algorithms for the evacuation of one source node to one designated destination (one-to-one evacuation). Besides, they also extend their model to solve one-to-many evacuations. Finally, case analysis of evacuation optimization and planning in Beijing is given and the efficiency of the proposed model is illustrated.

In the paper “*An improved hierarchical genetic algorithm for sheet cutting scheduling with process constraints,*” Y. Rao et al. proposed an improved hierarchical genetic algorithm for sheet cutting problem which involves *n* cutting patterns for *m* nonidentical parallel machines with process constraints. Furthermore, to speed up convergence rates and resolve local convergence issues, a kind of adaptive crossover probability and a kind of mutation probability are used in this algorithm. The computational result and comparison prove that the presented approach is quite effective for the considered problem.

In the paper entitled “*An adaptive hybrid algorithm based on particle swarm optimization and differential evolution for global optimization*,” X. Yu et al. formulate a novel adaptive hybrid algorithm based on PSO and DE (HPSO-DE) by developing a balanced parameter between PSO and DE. Adaptive mutation is carried out to current population when the population clusters around local optima. The HPSO-DE enjoys the advantages of PSO and DE and maintains diversity of the population. Compared with PSO, DE, and their variants, the performance of HPSO-DE is promising and competitive.

In the paper “*An effective hybrid self-adapting differential evolution algorithm for the joint replenishment and location-inventory problem in a three-level supply chain*,” L. Wang et al. provide an effective intelligent algorithm for a modified joint replenishment and location-inventory problem (JR-LIP) where distribution centers (DCs) replenish their demands jointly. The problem of the JR-LIP is to determine the number and locations of DCs to be opened, the assignment of retailers to DCs, the basic replenishment time of DCs, and the replenishment frequency of each DC such that the overall cost is minimized. To find an effective approach for the JR-LIP, a hybrid self-adapting differential evolution algorithm (HSDE) is designed. Comparative results of benchmark functions' tests and randomly generated JR-LIPs show that HSDE outperforms GA and HDE.

In the paper entitled “*Genetic algorithm application in optimization of wireless sensor networks*,” A. Norouzi and A. H. Zaim use genetic algorithm to optimize wireless sensor networks. A fitness function with optimum formula was obtained and the present protocols are optimized. The results of simulations in JPAC, MATLAB, and NS are compared with that of the present protocols and optimization of the two parameters is confirmed. It is also noticeable that the diagrams obtained from the simulations show an improvement in energy consumption parameters and lifetime of the network; this means more ideal WSNs.

In the paper, the research of X. Cheng and Y. Lin entitled “*Multiobjective robust design of the double wishbone suspension system based on particle swarm optimization*” proposes a robust design based on bioinspired computation. The simulation experiment is arranged and Latin hypercube design is adopted to find the initial point. Then sensitivity analysis is utilized to determine main design variables. The kriging model is employed for fitting the mean and variance of the quality characteristics according to the simulation results. Furthermore, a particle swarm optimization method based on simple PSO is applied and the tradeoff between the mean and deviation of performance is made to solve the robust optimization problem of double wishbone suspension system.

In the paper entitled “*A novel artificial bee colony approach of live virtual machine migration policy using Bayes theorem*,” G. Xu et al. present a novel heuristic approach which is called PS-ABC. Its algorithm includes two parts. One is that it combines the ABC (artificial bee colony) idea with the uniform random initialization idea and the binary search idea and Boltzmann selection policy to achieve an improved ABC-based approach with better global exploration's ability and local exploitation's ability. The other one is that it uses the Bayes Theorem to further optimize the improved ABC-based process to faster get the final optimal solution. As a result, the whole approach achieves a longer-term efficient optimization for power saving. The experimental results demonstrate that PS-ABC evidently reduces the total incremental power consumption and better protects the performance of VM running and migrating compared with the existing research. It makes the result of live VM migration more high effective and meaningful.

In the paper entitled “*Seven-spot ladybird optimization: a novel and efficient metaheuristic algorithm for numerical optimization*,” P. Wang et al. present a novel biologically inspired metaheuristic algorithm called seven-spot ladybird optimization (SLO). The SLO is inspired by recent discoveries on the foraging behavior of a seven-spot ladybird. In this paper, the performance of the SLO is compared with that of the genetic algorithm, particle swarm optimization, and artificial bee colony algorithms by using five numerical benchmark functions with multimodality. The results show that SLO has the ability to find the best solution with a comparative small population size and is suitable for solving optimization problems with lower dimensions.

In the paper entitled “*Pricing resources in LTE networks through multiobjective optimization,*” Y.-L. Lai and J.-R. Jiang study the pricing resources with profits and satisfaction optimization (PRPSO) problem in the LTE networks, considering the operator profit, and subscribe satisfaction at the same time. The problem is modeled as nonlinear multiobjective optimization with two optimal objectives: (1) maximizing operator profit and (2) maximizing user satisfaction. They propose solving the problem based on the framework of the NSGA-II algorithm. Simulations are conducted for evaluating the proposed solution.

In the paper entitled “*Comparison of multiobjective evolutionary algorithms for operations scheduling under machine availability constraints*,” M. Frutos et al. analyze different evolutionary multiobjective algorithms (MOEAs) for this kind of problems. They consider an experimental framework in which they schedule production operations for four real-world Job-Shop contexts using three algorithms, NSGAII, SPEA2, and IBEA. Using two performance indexes, hypervolume and R2, they found that SPEA2 and IBEA are the most efficient for the tasks at hand. On the other hand IBEA seems to be a better choice of tool since it yields more solutions in the approximate Pareto frontier.

In the paper entitled “*A modified decision tree algorithm based on genetic algorithm for mobile user classification problem*,” D.-S. Liu and S.-J. Fan put forward a modified decision tree algorithm for mobile user classification, which introduced genetic algorithm to optimize the results of the decision tree algorithm. They also take the context information as a classification attributes for the mobile user and classify the context into public context and private context classes. Then, the processes and operators of the algorithm are analyzed. At last, an experiment is given so as to verify the efficiency and effectiveness of the proposed approach.

In the paper “*A location selection policy of live virtual machine migration for power saving and load balancing*,” J. Zhao et al. present the specific design and implementation of MOGA-LS such as the design of the genetic operators and fitness values and elitism. They introduce the Pareto dominance theory and the SA (simulated annealing) idea into MOGA-LS and present the specific process to get the final solution. And thus the whole approach achieves a long-term efficient optimization for power savings and load balancing. The experimental results demonstrate that MOGA-LS evidently reduces the total incremental power consumption, better protects the performance of VM migration, achieves the balancing of system load compared with the existing research. It makes the result of live VM migration more high effective and meaningful.

In the paper “*Modeling Markov switching ARMA-GARCH neural networks models and an application to forecasting stock returns,*” M. E. Bildirici and O. Ersin propose a family of regime switching GARCH neural network models to model volatility. Proposed MS-ARMA-GARCH-NN models allow MS type regime switching in both the conditional mean and conditional variance for time series and further augmented with artificial neural networks to achieve improvement in forecasting capabilities. In the empirical section, daily stock returns in ISE100 Istanbul Stock Index are modeled and forecasted. Forecast success is evaluated with MAE, MSE, and RMSE criteria and Diebold-Mariano tests. Result suggest that hybrid MLP and time lag recurrent MS-ARMA-FIAPGARCH hybrid MLP and MS-ARMA-FIAPGARCH-RNN provided the best forecast and modeling performance over the simple GARCH models and additionally Gray's MS-GARCH model. Therefore, augmenting the forecasting capabilities with neural networks and regime switching provide significant gains in various economic applications.

In the paper entitled “*Full glowworm swarm optimization algorithm for whole-set orders scheduling in single machine*,” Z. Yu and X. Yang propose a new glowworm swarm optimization algorithm for scheduling by analyzing the characteristics of whole-set orders problem and combining the theory of glowworm swarm optimization. A new hybrid-encoding schema combining with two-dimensional encoding and random-key encoding is given. In order to enhance the capability of optimal searching and speed up the convergence rate, the dynamical changed step strategy is integrated into this algorithm. Furthermore, experimental results prove its feasibility and efficiency.

In the paper “*Multiple R&D scheduling optimization with improved particle swarm algorithm*,” M. Liu et al. discuss the features of multiple R&D environment in customization enterprises and demands of resources distribution, make some improvements to the multiple project scheduling models, and put forward a multiple project crashing scheduling model based on postpone-punishment in large scale customization environment. At the same time, based on the analysis of particle swarm optimization algorithm and its improvement, a new solution of dynamic center particle swarm optimization algorithm to multiproject scheduling model has been arisen. The best solution can be gained after the experiment to the illustration example through MATLAB program; thus, the model in the paper and application of algorithm can be proved.

In the paper “*Applying probability theory for the quality assessment of a wildfire spread prediction framework based on genetic algorithms*,” A. Cencerrado et al. present a framework for assessing how the existing constraints at the time of attending an ongoing forest fire affect simulation results, both in terms of quality (accuracy) obtained and the time needed to make a decision. The core of this framework is evaluated according to the probability theory principles. Thus, a strong statistical study is presented, oriented towards the characterization of such an adjustment technique in order to help the operation managers to deal with the two aspects previously mentioned: time and quality. The experimental work in this paper is based on a region in Spain which is one of the most prone to forest fires: El Cap de Creus.

In the paper “*Scheduling projects with multiskill learning effect*,” H. Zha and L. Zhang investigate the project scheduling problem with multiskill learning effect. A new model is proposed to deal with the problem, where both autonomous and induced learning are considered. In order to obtain the optimal solution, a genetic algorithm with specific encoding and decoding schemes is introduced. A numerical example is used to illustrate the proposed model. The computational results show that the learning effect cannot be neglected in project scheduling. By means of determining the level of induced learning, the project manager can balance the project makespan with total cost.

In the paper entitled “*A novel algorithm combining the finite state method and genetic algorithm for scheduling of crude oil problem*,” Q.-Q. Duan et al. propose a novel genetic algorithm to solve the MINLP problem. The MINLP model they discussed is based on the single-operation sequencing (SOS) time representation. In this paper, based on the sequencing rules and the extension to the regular expression calculus, a deterministic finite state automaton (DFA) which captures valid possible schedule sequences is constructed. In the initial population stage of GA, a population of candidate solutions is generated on the basis of this DFA. The rule-based mutation strategy consists in following the sequencing rule and meeting the nonoverlapping constraint, which is moving towards optimal solutions efficiently. The optimization results indicate both the effectiveness of the model and efficiency as well as the robustness of the solution methodology.

In the paper “*Improved particle swarm optimization with a collective local unimodal search for continuous optimization problems*,” M. A. Arasomwan and A. O. Adewumi propose a new local search technique and use it to improve the performance of particle swarm optimization algorithms by addressing the problem of premature convergence. In the proposed local search technique, a potential particle position in the solution search space is collectively constructed by a number of randomly selected particles in the swarm. The number of times the selection is made varies with the dimension of the optimization problem and each selected particle donates the value in the location of its randomly selected dimension from its personal best. After constructing the potential particle position, some local search is done around its neighborhood in comparison with the current swarm global best position. It is then used to replace the global best particle position if it is found to be better; otherwise, no replacement is made. Using some well-studied benchmark problems with low and high dimensions, numerical simulations are used to validate the performance of the improved algorithms. Comparisons are made with four different PSO variants; two variants implement different local search technique, while the other two do not. Results show that the improved algorithms could obtain better quality solution while demonstrating better convergence velocity and precision, stability, robustness, and global-local search ability than the competing variants.

In the paper “*A new collaborative recommendation approach based on users clustering using artificial bee colony algorithm*,” C. Ju and C. Xu propose a novel collaborative filtering recommendation approach based on* K*-means clustering algorithm. Firstly, they use artificial bee colony (ABC) algorithm to overcome* K*-means algorithm's problems. And then they adopt the modified cosine similarity considering products' popularity degrees and users' preference degrees to compute the similarity between users in the same clusters. Finally, they generate the recommendation results for target users. Detailed numerical analysis on a benchmark dataset* MovieLens* and a real-world dataset indicates that their new collaborative filtering approach based on users clustering algorithm outperforms many other recommendation methods.

In the paper “*Comprehensive optimization of emergency evacuation route and departure time under traffic control,*” G. Li et al. investigate the comprehensive optimization of major emergency evacuation route and departure time, in which evacuation propagation mechanism is considered under traffic control. Based on practical assumptions, they first establish a comprehensive optimization model based on the simulation of evacuation route and departure time. In order to optimize the evacuation routes and departure time, they explore the reasonable description methods of evacuation traffic flow propagation under traffic control, including the establishment of traffic flow propagation model and the design of algorithm which can implement simulation of evacuation traffic flow. Finally, they propose a heuristic algorithm for optimization of this comprehensive model. In case analysis, they take some areas in Beijing as evaluation sources to verify the reliability of this model. Moreover, some constructive suggestions for Beijing's emergency evacuation are proposed, which can be applied to actual situation, especially under traffic control.

In the paper “*Collaborative scheduling model for Supply-Hub with multiple suppliers and multiple manufacturers*,” G. Li et al. investigate a collaborative scheduling model that contains multiple suppliers, multiple manufacturers, and a Supply-Hub. They describe the operational process of Supply-Hub and formulate the basic scheduling model. Based on this, they consider two different scenarios: one is that the suppliers and the manufacturers make their decisions separately and the other is that Supply-Hub makes the entire decisions with the collaborative scheduling. Under this condition, they prove that the scheduling model with Supply-Hub is NP-complete issue; thus, an autoadapted differential evolution algorithm is proposed. In the numerical analysis, they illustrate that the performance of collaborative scheduling for Supply-Hub is superior to separate decision made by each manufacturer and supplier. Furthermore, they also show that the proposed algorithm has a good convergence and reliability, in particular when it can be exerted flexibly onto certain suppliers.

In the paper entitled “*Hierarchical artificial bee colony algorithm for RFID network planning optimization*,” L. Ma et al. present an optimization model for planning the positions and radiated power setting of readers in the RFID network. The four nonlinear RNP objective functions are formulated with considering tag coverage, reader interference, economic efficiency, and network load balance as the primary requirements of the real-world RFID system. And the combined measure is also given so that the multiple objectives can be optimized simultaneously. Finally, in order to solve the RNP model, a novel hierarchical artificial colony algorithm, called HABC, is proposed by extending single artificial bee colony (ABC) algorithm to hierarchical and cooperative mode by combining the multipopulation cooperative coevolution approach based on vector decomposing strategy and the comprehensive learning method. Results obtained from the proposed approach have been compared with those obtained by ABC, PSO, CPSO, EGA, CMA_ES, and CCEA. The experiment results show that, for all the test functions, the HABC gets significant superiority to other six algorithms. HABC is then employed to solve the real-world RNP problem on two different-scale instances, namely, Cd100 and Rd500. Through simulation studies, the HABC remarkably outperforms other algorithms. Especially, in tackling larger-scale RNP problem (i.e., Rd500 instance), the HABC performs more effectively, indicating that the HABC is more suitable for solving high-dimension RNP problem.

The study of bioinspired computation and its applications in operation management is still in its early stage. This special issue demonstrates the theoretical and practical importance of further studies on bioinspired computation.

